# Robotic-assisted versus conventional minimally invasive esophagectomy: a retrospective cohort study from a high-volume center

**DOI:** 10.1007/s11701-025-02590-0

**Published:** 2025-07-22

**Authors:** Fahad Murad, Francesca Blasa, Daniela Polette Stubb, Mats Lindblad, Fredrik Klevebro, Chih-Han Kung, Ioannis Rouvelas

**Affiliations:** 1 Department of Upper Abdominal Surgery, Center for Digestive Diseases, Karolinska University Hospital, Huddinge, 141 86 Stockholm, Sweden; 2 Division of Surgery and Oncology, Department of Clinical Science, Intervention and Technology (CLINTEC), Karolinska Institutet, Huddinge, C177, 141 86 Stockholm, Sweden

**Keywords:** Esophageal cancer, Robotic esophagectomy, Minimally invasive esophagectomy, Ivor-Lewis esophagectomy

## Abstract

As a newer surgical technique, robotic-assisted minimally invasive esophagectomy (RAMIE) offers an alternative to conventional minimally invasive esophagectomy (cMIE) in the treatment of esophageal cancer. The objective of this study was to evaluate and compare postoperative outcomes of RAMIE versus cMIE in patients undergoing Ivor-Lewis esophagectomy at a high-volume institution. The medical records from patients who underwent minimally invasive Ivor-Lewis esophagectomy between 2015 and 2024 were retrospectively analyzed. Data on surgical and oncological outcomes, such as conversions to open surgery, lymph node yield, anastomotic leakage rates, postoperative complications, and mortality, were collected and evaluated. The analysis included a total of 250 patients: 170 underwent cMIE, and 80 underwent RAMIE. Patient baseline characteristics were comparable across both groups, except for differences in neoadjuvant therapy use. Importantly, no mortality was recorded within 30- or 90-day following RAMIE, and conversion rates were low in both cohorts. The rates of anastomotic leaks, major postoperative complications, and harvested lymph nodes were comparable across the two groups. Additionally, multivariable logistic regression analysis controlling for potential confounders confirmed that RAMIE and cMIE had similar leak rates. The current findings indicate that RAMIE represents a safe, feasible option comparable to cMIE, with potential ergonomic advantages and improved postoperative recovery. However, additional studies in larger groups will be necessary to validate these findings and determine long-term oncologic outcomes.

**Supplementary Information:**

The online version contains supplementary material available at 10.1007/s11701-025-02590-0.

## Introduction

Esophageal cancer remains among the most aggressive malignancies globally, accounting for over 600,000 new diagnoses and approximately 540,000 deaths in 2020 [1]. Over recent decades, minimally invasive esophagectomy (MIE) has established itself as the preferred surgical approach for managing esophageal cancer [2]. Although technically challenging with a significant learning curve [3], MIE has demonstrated clear benefits over open esophagectomy, including reduced intraoperative blood loss, shorter hospitalization, fewer pulmonary and cardiovascular complications, and decreased in-hospital mortality [4, 5]. Ivor-Lewis esophagectomy is especially challenging due to the intrathoracic anastomosis. Prior evidence suggests that MIE offers superior perioperative results and lower rates of in-hospital mortality relative to open esophagectomy. (OE) [6–9]. However, many of these studies are from Asia, most of which include cervical anastomosis. Despite that factor, there are comparable results for conventional MIE (cMIE) versus open Ivor-Lewis esophagectomy [10].

The recent use of robotic-assisted MIE (RAMIE) has shown this technique to facilitate dissection with potential advantages in precision, visualization, and surgical ergonomics. A recent multicenter randomized controlled trial comparing RAMIE McKeown with cMIE reported enhanced lymph node retrieval near the recurrent laryngeal nerve during RAMIE, resulting in fewer nerve-related injuries [11]. The intrathoracic anastomosis remains among the most technically challenging aspects of Ivor-Lewis esophagectomy; robotic assistance can significantly facilitate this step by providing enhanced precision during dissection within the confined space of the posterior mediastinum [12]. The ROBOT trial, which compared RAMIE McKeown with open esophagectomy, demonstrated that RAMIE resulted in fewer postoperative complications, reduced postoperative pain, improved functional recovery, and enhanced short-term quality of life, while achieving similar oncological outcomes [13]. However, data on the comparison between RAMIE Ivor-Lewis and cMIE, excluding single-center studies, is scarce. The preliminary findings from a randomized trial by Yang et al. indicated that RAMIE may offer reduced operation time and enhanced lymph node retrieval in neoadjuvantly treated patients with esophageal squamous cell carcinoma (ESCC). However, long-term outcomes from this study have not yet been reported [14].

This study aims to compare RAMIE to MIE in Ivor-Lewis esophagectomy regarding anastomotic leak rate and postoperative complications in a high-volume, single-center setting.

## Methods

### Study design and definition of exposure

This is a single-center cohort study of all patients operated on with Ivor-Lewis MIE from January 2015 to December 2024. The introduction of MIE at our institution was in 2011, and our results have been previously published [15]. The introduction of RAMIE followed the standard process of robotic implementation with console training and proctoring between 2020 and 2021, with some interruption due to the COVID-19 pandemic. Thus, RAMIE was performed from 2021, and the first five cases of RAMIE without proctoring were excluded as this was considered as introduction phase for this institution. Both cMIE and RAMIE were carried out using a standardized technique, employing a minimally invasive approach for both the abdominal and thoracic phases.

All patients scheduled for minimally invasive thoracic surgery were included in the analysis, regardless of whether a hybrid abdominal approach or conversion to open surgery in the abdominal phase occurred.

All procedures, both RAMIE and cMIE, were performed by the same four surgeons, all of whom were proficient in cMIE before the introduction of RAMIE. There were no patient-related factors or surgeon-related factors for the selection process for RAMIE or cMIE. Since the introduction of RAMIE, the institutional protocol is to perform all minimally invasive Ivor-Lewis esophagectomies as RAMIE. The unit has access to two theater days per week with the DaVinci^®^ Xi system, and any cMIE after the robotic implementation is due to the unavailability of the robotic system. cMIE was performed with 3D full HD laparoscopy/thoracoscopy with patients in the chest phase in prone position with four ports in the chest. RAMIE procedures were conducted with the patient in a semi-prone position, utilizing three robotic ports and two assistant ports. The gastric conduit was prepared intracorporeally in a standard fashion and stapled with Endo-Gia^®^ Tri-Staple purple cartridge for cMIE and Sureform^®^ blue cartridge for RAMIE. All anastomoses were constructed using a side-to-side technique with linear staplers: the Endo-GIA^®^ Tri-Staple brown cartridge was used for cMIE, while the EndoWrist^®^ blue cartridge was employed in RAMIE procedures. In both groups, the stapler entry site was closed manually using full-thickness 4/0 absorbable V-Loc^®^ sutures, followed by placement of an omental wrap around the anastomosis. The perioperative care is standardized and the same for cMIE and RAMIE according to the unit’s enhanced recovery protocol.

### Data source

All patients were recruited through the institutional database record of all esophagectomies performed by this institution. Data were collected from patients’ electronic medical records.

### Definition of outcomes

The primary endpoint was the occurrence of anastomotic leaks within 30 days, classified as grade 2 or higher according to the Esophagectomy Complications Consensus Group (ECCG) criteria. Secondary endpoints included lymph node yield and overall morbidity, defined as Clavien–Dindo grade 3 or above. Mortality at 30 and 90 days, as well as conversion to open surgery, were also assessed.

### Statistics

Data are reported as mean (standard deviation) or median (interquartile range), as appropriate. The categorical variables were compared using the Chi-square test, while continuous variables were analyzed with the Student’s t-test. Fisher’s exact test was applied to directly compare 30- and 90-day mortality rates between the two groups. To estimate the odds ratio (OR) of anastomotic leak grade 2 or higher between the two study groups, logistic regression was performed with a multivariable model adjusting for age (continuous), gender (male/female), clinical tumor-node-metastasis staging (cTNM8 Stage I–IV), type of neoadjuvant treatment and the American Society of Anesthesiologists (ASA) physical status classification (grades I–IV). Results are reported as odds ratios (ORs) with corresponding 95% confidence intervals (CIs). A p-value ≥ 0.05 was considered statistically significant. All statistical analyses were conducted using SPSS version 29 (IBM, Armonk, NY).

### Ethics

Ethical approval for this study was obtained from the Regional Ethics Review Board in Stockholm (EPN), under the following reference numbers: 2018/970–31/1, 2020–01459, 2021–02810, 2023–02802-02, and 2024–03122-02.

## Results

**Fig. 1:**
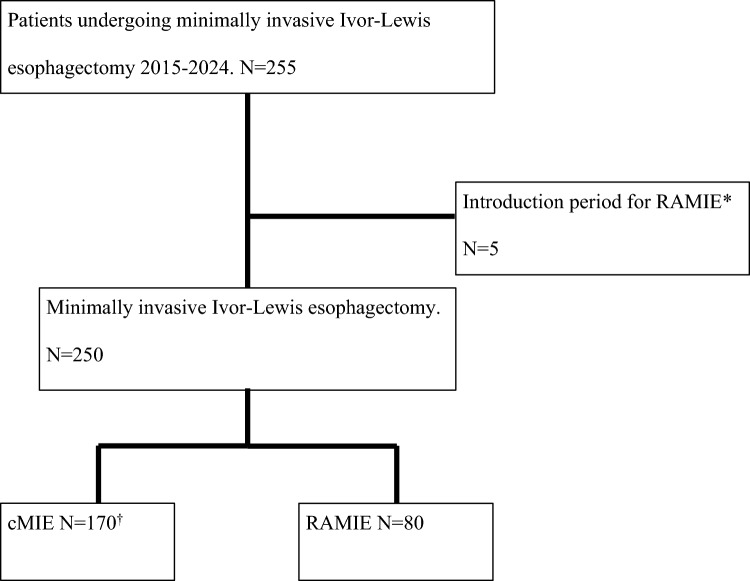
Flow chart of patient selection and inclusion. *RAMIE: Robot-Assisted Minimally Invasive Esophagectomy. †cMIE. Conventional Minimally Invasive Esophagectomy

Between 2015 and 2024, a total of 170 patients underwent MIE and 80 patients received RAMIE. A flow chart of study selection can be seen in Fig. 1.

The median age in both groups was 69 years (33–83). Adenocarcinoma was the predominant histological subtype, with only 11 cases of squamous cell carcinoma—9 in the cMIE group and 2 in the RAMIE group. In both groups, the gastroesophageal junction was the most common tumor location. In general, the most frequent clinical stage was III, followed by stage IV, II, and I. Regarding the multimodal therapy, it is important to note that in the RAMIE group, there were more patients with preoperative chemotherapy, while in the cMIE group, it was more frequent to undergo neoadjuvant chemoradiotherapy. The RAMIE group had a higher proportion of patients with better Eastern Cooperative Oncology Group (ECOG) performance status, although a greater proportion of patients in this group also had ASA score III compared with the cMIE group, indicating a higher burden of comorbidities. Additional baseline characteristics are summarized in Table 1.

**Table 1:** Baseline demographic and tumor characteristics of patients undergoing conventional minimally invasive esophagectomy (cMIE) and robot-assisted minimally invasive esophagectomy (RAMIE)

	cMIE (*n* = 170)	RAMIE (*n* = 80)	*p* value
Age, median (range)	69 (33–83)	69 (33–83)	0.545
Gender			0.346
Female	24 (14.1)	15 (18.8)	
Male	146 (85.9)	65 (81.3)	
^‡^BMI, mean (SD)	26.3 (4.4)	26.1 (4.0)	0.711
*Performance Status			< 0.001
0	66 (38.8)	61 (76.3)	
1	94 (55.3)	17 (21.3)	
2	4 (2.4)	1 (1.3)	
Missing	6 (3.5)	1 (1.3)	
^†^ASA-score			< 0.001
I	53 (31.2)	0	
II	82 (48.2)	33 (41.3)	
III	35 (20.6)	46 (57.5)	
IV	0	1 (1.3)	
Histologic type			0.315
Adenocarcinoma	161 (94.7)	78 (97.5)	
Squamous cell carcinoma	9 (5.3)	2 (2.5)	
Tumor location			< 0.001
Middle esophagus	7 (4.1)	1 (1.3)	
Distal esophagus	29 (17.1)	33 (41.3)	
Gastroesophageal junction	134 (78.8)	46 (57.5)	
Clinical stage TNM8			0.596
I	6 (3.5)	1 (1.3)	
II	16 (9.4)	5 (6.3)	
III	110 (64.7)	54 (67.5)	
IV	38 (22.4)	20 (25.0)	
Multimodal therapy			< 0.001
Surgery alone	38 (22.4)	9 (11.3)	
Perioperative chemotherapy	44 (25.9)	63 (78.8)	
Neoadjuvant chemoradiotherapy	88 (51.8)	8 (10.0)	

Percentages are indicated in parentheses, unless otherwise specified

*ECOG Eastern cooperative oncology group, ^†^*ASA* American society of anesthesiologists, ^‡^*BMI* body mass index

In terms of surgical outcomes, the conversion rate was below 1.8% for the cMIE group and 0% for the RAMIE group; however, this difference was not statistically significant. Operative time was longer in the RAMIE group, while intraoperative blood loss was comparable between the groups, while length of hospital stay was marginally shorter in the RAMIE cohort. The comprehensive operative details are provided in Table 2.

**Table 2:** Operative and short-term postoperative outcomes in patients undergoing conventional minimally invasive esophagectomy (cMIE) and robot-assisted minimally invasive esophagectomy (RAMIE)

	cMIE (*n* = 170)	RAMIE (*n* = 80)	*p*-value
Operation time, mean min (SD)	418 (82)	519 (86)	< 0.001
Perioperative blood loss, mean ml (SD)	178 (267)	194 (263)	0.653
Hospital stay, median days (range)	13 (6–148)	10 (6–97)	0.037
Conversion chest	1 (0.6)	0	1*
Conversion abdomen	3 (1.8)	0	0.553*
Clavien–Dindo score			< 0.001
0–I	55 (32.4)	32 (40.0)	
II	46 (27.1)	20 (25.0)	
IIIa	31 (18.2)	6 (7.5)	
IIIb	18 (10.6)	16 (20.0)	
IVa	11 (6.5)	5 (6.3)	
IVb	1 (0.6)	1 (1.3)	
V	8 (4.7)	0	
Anastomotic Leak *ECCG type II or higher	41 (24.1)	15 (18.8)	0.342
Stent or esosponge for leak	35 (20.6)	15 (18.8)	
Number of resected lymph nodes, mean (SD)	37 (16)	38 (12)	0.436
R0 resection	163 (95.9)	72 (90.0)	0.110
Pathological stage TNM8			0.219
Stage 0, (ypT0N0)	28 (16.5)	6 (7.5)	
I	33 (19.4)	13 (16.3)	
II	42 (24.7)	19 (23.8)	
III	40 (23.5)	24 (30.0)	
IV	27 (15.8)	18 (22.5)	
30-day mortality	5 (2.9)	0	0.180
90-day mortality	9 (5.3)	0	0.061

Percentages are indicated in parentheses, unless otherwise specified

*ECCG Esophagectomy Complications Consensus Group

Anastomotic leak occurred in 24.1% of patients in the cMIE group and 18.8% in the RAMIE group. Re-interventions for leaks, such as placement of an Esosponge or stent, were needed in 20.6% and 18.8% of cases, respectively. Notably, no patient required complete conduit removal. The number of lymph nodes retrieved was similar across both groups. No significant differences were observed in 30- or 90-day mortality between the cohorts.

Clavien–Dindo grade III or higher complications occurred in both groups. A detailed breakdown of these complications is provided in Supplementary Table 1. No statistically significant differences were observed between groups.

Table 3 displays the odds ratio (OR) for anastomotic leak. Multivariable logistic regression analysis revealed no significant difference in leak rates between the groups, with an OR of 0.772 (95% CI: 0.327–1.822; *p* = 0.554) for RAMIE versus cMIE. Due to the absence of deaths in the RAMIE group, ORs for 30- and 90-day mortality could not be computed. To address this, a sensitivity analysis was conducted in which one RAMIE patient who experienced severe postoperative complications (Clavien–Dindo grade IVb) and died on postoperative day 117 was reclassified as a 30- and 90-day mortality event. This reclassification had no meaningful impact on the results, and the difference in mortality between groups remained statistically non-significant.

**Table 3:** Multivariable logistic regression analysis for risk of anastomotic leak, major complications, and 30- and 90-day mortality

	cMIE	RAMIE OR (95% CI)	*p* value
Anastomotic leak			
Crude	Reference	0.726 (0.374–1.408)	0.344
*Adjusted	Reference	0.772 (0.327–1.822)	0.554
CD III or higher			
Crude	Reference	0.788 (0.454–1.369)	0.398
Adjusted	Reference	0.944 (0.452–1.970)	0.877
30-day mortality			
Crude	Reference	N/A^†^	0.997
Adjusted	Reference	N/A^†^	0.996
Adjusted Sensitivity^‡^	Reference	1.029 (0.098–10.853)	0.981
90-day mortality			
Crude	Reference	N/A†	0.997
Adjusted	Reference	N/A†	0.996
Adjusted Sensitivity^**‡**^	Reference	0.484 (0.051–4.565)	0.526

*The model is adjusted for age, gender, clinical tumor stage, type of neoadjuvant treatment, and American Society of Anesthesiologists (ASA); ^†^Exact odds ratios for 30- and 90-day mortality could not be reliably estimated due to the absence of events in the RAMIE group, resulting in unstable maximum likelihood estimates; ^‡^A sensitivity analysis was performed by reclassifying as 30- and 90-day mortality a RAMIE patient who experienced severe postoperative complications (Clavien–Dindo IVb) and died on postoperative day 117

Supplementary Fig. 1 illustrates the learning curves observed in the RAMIE group. Operative time, anastomotic leak rates (ECCG grade ≥ 2), lymph node yield, and blood loss were plotted across consecutive cases in 10-case intervals. These data reflect the progression of technical proficiency and procedural standardization achieved within a relatively short timeframe following the implementation of the robotic program.

## Discussion

This single-center cohort study evaluated short-term outcomes following Ivor-Lewis esophagectomy using either RAMIE or cMIE. Our results suggest that RAMIE is a safe and viable surgical approach, yielding outcomes that are at least comparable to those of cMIE. Notably, the absence of conversions to open surgery and the lack of 30- and 90-day postoperative mortality in the RAMIE group point toward a potential advantage in perioperative safety.

The primary outcome, anastomotic leak rate, was lower in the RAMIE group (18.8%) than in the cMIE group (24.1%), though this difference did not reach statistical significance. Multivariable logistic regression, accounting for baseline and treatment-related variables, also demonstrated no significant difference in leak rates. Likewise, the incidence of major postoperative complications and the duration of hospital stay were similar between the two groups. Lymph node yield was also similar between groups, suggesting equivalent oncological completeness regardless of surgical approach.

Although patients in the RAMIE group had a more favorable ECOG performance status, a higher proportion also had ASA score III compared with the cMIE group, indicating a comparable or even greater comorbidity burden. Additionally, the median age was identical in both groups, making age an unlikely explanation for any observed differences in outcomes.

Operative time was longer in the RAMIE group, which likely reflects the early phase of robotic program adoption and learning. Our results are consistent with recent meta-analyses comparing RAMIE and cMIE, which collectively support RAMIE as a safe and feasible alternative offering similar perioperative outcomes. Of note, RAMIE has been linked to fewer pulmonary complications, increased lymph node retrieval, and trends toward reduced blood loss and shorter hospitalization, underscoring its potential technical and clinical advantages, even during early implementation [16–18]. These findings are further supported by large comparative cohort studies, such as Tagkalos et al., which showed similar or improved perioperative outcomes with RAMIE in a propensity-matched analysis [19]. Randomized controlled trials further support these advantages, reporting improved lymph node dissection and reduced nerve injury with RAMIE [11, 14].

It is also noteworthy that these results were achieved with RAMIE over a relatively short timeframe following the program’s implementation, suggesting a rapid and effective learning curve. This aligns with prior reports indicating that the structured adoption of robotic platforms can lead to faster proficiency compared to cMIE, particularly in technically complex procedures such as Ivor-Lewis esophagectomy [3, 20]. Although CUSUM analysis was not performed due to a lack of detailed case-by-case event data, learning curve trends were illustrated by plotting key metrics in 10-case intervals.

A key strength of this study lies in the consistent application of surgical techniques and standardized perioperative management across all cases, supported by a structured enhanced recovery protocol applied equally to both groups. All surgeries were performed by the same four surgeons, all of whom were experienced in cMIE before transitioning to RAMIE. This consistency minimizes inter-surgeon variability and strengthens internal validity.

However, several limitations must be acknowledged. As a retrospective study, this analysis is subject to inherent bias. Additionally, a higher proportion of patients in the cMIE group received neoadjuvant chemoradiotherapy, a factor previously linked to an elevated risk of anastomotic complications, which may have influenced the comparison. Although our study spans from 2015 to 2024 and reflects contemporary practice, it also captures a transition period in neoadjuvant treatment strategy, from chemoradiotherapy to perioperative chemotherapy, which may limit the generalizability of the findings. While multivariable adjustments were applied, the possibility of residual confounding remains. Moreover, the relatively limited sample size, particularly in the RAMIE cohort, reduces the statistical power to detect subtle differences, especially in rare events such as mortality. Finally, operative time was reported as a total value, as phase-specific durations (abdominal vs thoracic) were not consistently recorded, which limits a more detailed analysis.

Future research should prioritize prospective, multicenter studies designed to evaluate the long-term oncological efficacy and economic viability of RAMIE compared to cMIE. Additionally, the integration of emerging technologies such as indocyanine green (ICG) fluorescence imaging and artificial intelligence (AI)-driven surgical guidance holds promise for enhancing operative precision and should be the focus of future trials [21, 22].

In conclusion, this study provides further evidence that RAMIE is both a safe and practical option in the surgical management of esophageal cancer. While outcomes were comparable to cMIE, the complete absence of mortality and zero conversions in the RAMIE group is promising. RAMIE may offer advantages in precision and ergonomics; however, the study is underpowered to definitively demonstrate superiority. Despite the growing body of comparative data, our findings provide additional insight into the early adoption of RAMIE within a structured, high-volume program, reinforcing its feasibility and safety in routine clinical practice. Larger prospective trials are warranted to validate these findings.

## Supplementary Information

Below is the link to the electronic supplementary material.

Supplementary file1 (DOCX 73 KB)

## Data Availability

Data are provided upon request.
