# Supplementary material for: Robotic-assisted versus conventional minimally invasive esophagectomy: a retrospective cohort study from a high-volume center
Source: J Robot Surg. 2025 Jul 22;19(1):414. doi: 10.1007/s11701-025-02590-0 (PMC12283888; doi:10.1007/s11701-025-02590-0)
Supplement: Supplementary file 1 — Supplementary file1 (DOCX 73 KB) [file 11701_2025_2590_MOESM1_ESM.docx]

**Supplementary Table 1.***Postoperative complications graded Clavien–Dindo ≥ III in patients undergoing conventional Minimally Invasive Esophagectomy (cMIE) and* robotic-assisted minimally invasive esophagectomy *(RAMIE).*

|  | cMIE (n=170) | RAMIE (n=80) | p-value |
| --- | --- | --- | --- |
| Anastomotic Leak *ECCG type II or higher | 41 (24.1) | 15 (18.8) | 0.342 |
| Airway Fistula | 3 (1.8) | 3 (3.8) | 0.388 † |
| Organ Failure | 20 (11.8) | 6 (7.5) | 0.378 † |
| Respiratory failure | 18 (10.6) | 4 (5.0) | 0.146 |
| Postop bleeding | 1 (0.6) | 0 (0) | 1.000 † |
| ^‡^DGCE/pyloric stenosis | 3 (1.8) | 3 (3.8) | 0.388 † |
| Paraconduit herniation | 5 (2.9) | 0 (0) | 0.180 † |

Complications classified as Clavien–Dindo grade III or higher. Data are presented as the number of patients (%). *****ECCG = Esophagectomy Complications Consensus Group; †Fisher’s Exact Test was used when appropriate; ^‡^DGCE = Delayed Gastric Conduit Emptying.

**Supplementary Figure 1**. Learning curve across consecutive robotic-assisted minimally invasive esophagectomy (RAMIE) cases per 10-case interval. *(A) Operative time (minutes), (B)Anastomotic leak, (C) number of lymph nodes retrieved, and (D) blood loss (ml). Trends reflect procedural standardization and learning over time.*

**A**


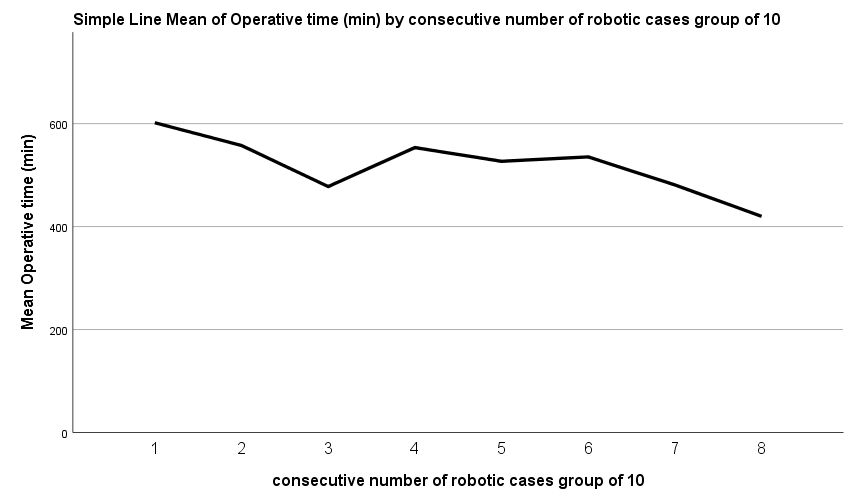


**B**


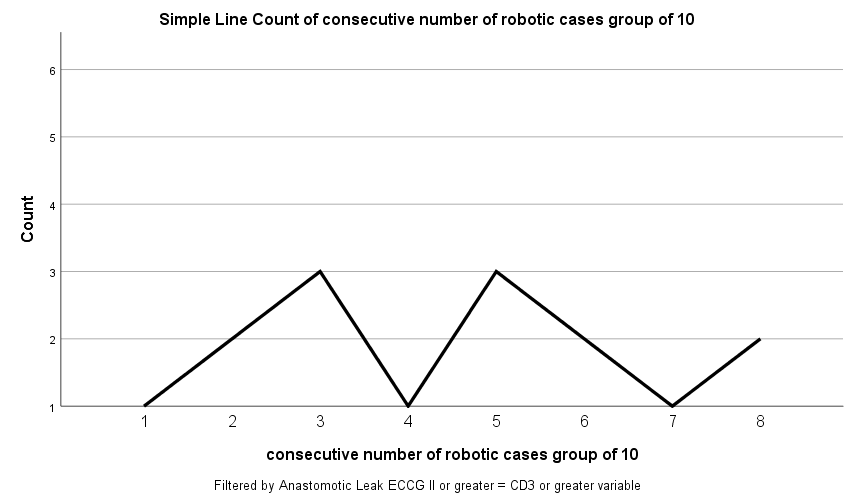


**C**


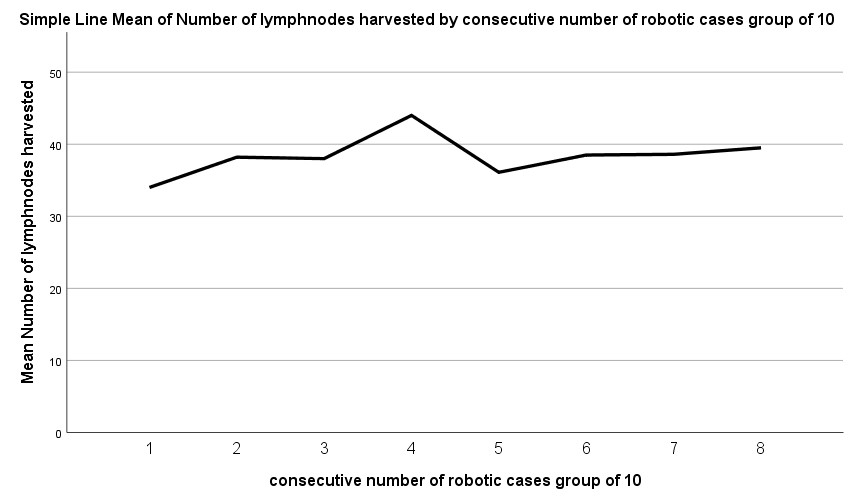


**D**
